# The Influence of Na^+^, K^+^-ATPase on Glutamate Signaling in Neurodegenerative Diseases and Senescence

**DOI:** 10.3389/fphys.2016.00195

**Published:** 2016-06-02

**Authors:** Paula F. Kinoshita, Jacqueline A. Leite, Ana Maria M. Orellana, Andrea R. Vasconcelos, Luis E. M. Quintas, Elisa M. Kawamoto, Cristoforo Scavone

**Affiliations:** ^1^Department of Pharmacology, Institute of Biomedical Science, University of São PauloSão Paulo, Brazil; ^2^Laboratory of Biochemical and Molecular Pharmacology, Institute of Biomedical Sciences, Federal University of Rio de JaneiroRio de Janeiro, Brazil

**Keywords:** Na^+^, K^+^-ATPase, glutamate, aging, *ATP1A2* and *ATP1A3* mutations, neurodegenerative diseases

## Abstract

Decreased Na^+^, K^+^-ATPase (NKA) activity causes energy deficiency, which is commonly observed in neurodegenerative diseases. The NKA is constituted of three subunits: α, β, and γ, with four distinct isoforms of the catalytic α subunit (α_1−4_). Genetic mutations in the *ATP1A2* gene and *ATP1A3* gene, encoding the α_2_ and α_3_ subunit isoforms, respectively can cause distinct neurological disorders, concurrent to impaired NKA activity. Within the central nervous system (CNS), the α_2_ isoform is expressed mostly in glial cells and the α_3_ isoform is neuron-specific. Mutations in *ATP1A2* gene can result in familial hemiplegic migraine (FHM2), while mutations in the *ATP1A3* gene can cause Rapid-onset dystonia-Parkinsonism (RDP) and alternating hemiplegia of childhood (AHC), as well as the cerebellar ataxia, areflexia, pescavus, optic atrophy and sensorineural hearing loss (CAPOS) syndrome. Data indicates that the central glutamatergic system is affected by mutations in the α_2_ isoform, however further investigations are required to establish a connection to mutations in the α_3_ isoform, especially given the diagnostic confusion and overlap with glutamate transporter disease. The age-related decline in brain α_2∕3_ activity may arise from changes in the cyclic guanosine monophosphate (cGMP) and cGMP-dependent protein kinase (PKG) pathway. Glutamate, through nitric oxide synthase (NOS), cGMP and PKG, stimulates brain α_2∕3_ activity, with the glutamatergic N-methyl-D-aspartate (NMDA) receptor cascade able to drive an adaptive, neuroprotective response to inflammatory and challenging stimuli, including amyloid-β. Here we review the NKA, both as an ion pump as well as a receptor that interacts with NMDA, including the role of NKA subunits mutations. Failure of the NKA-associated adaptive response mechanisms may render neurons more susceptible to degeneration over the course of aging.

## Introduction

### Na^+^, K^+^-ATPase

In 1957, the Danish physician Jens C. Skou discovered the mechanism behind active ion transport in homogenates of leg nerve from shore crabs: a Mg^2+^-dependent ATPase stimulated by Na^+^ and K^+^, speculated to be located at the plasma membrane (Skou, 1957). In the same year that Skou published his ATPase work, Robert L. Post and Philip Jolly showed that interdependent active Na^+^ efflux and K^+^ influx followed an electrogenic stoichiometry of 3:2 (Post and Jolly, 1957), an exchange previously shown to be blocked by the cardiotonic steroid (CTS) isolated from plant species of the genus *Strophanthus*, strophantin, widely known as ouabain (OUA) (Schatzmann, 1953; Schatzmann and Witt, 1954). In 1960, Post and colleagues demonstrated the presence of Na^+^, K^+^-ATPase (NKA) in the plasma membrane of human red blood cells (Post et al., 1960), including its transient phosphorylation by ATP on an aspartyl residue (Post et al., 1965; Post and Kume, 1973). Subsequent work indicated a feed-forward reaction, whereby as Na^+^ binds to the enzyme, it stimulates a Mg^2+^-dependent phosphorylation via ATP, while K^+^ binding facilitates dephosphorylation, giving a transition of two conformations named E1 and E2, respectively (Post et al., 1972). Later work made it clear that activity of this molecular motor is vital for cell integrity, given its maintenance of the osmotic balance and its powerful role in cell homeostasis, including preserving and contributing to the resting membrane potential, electrolyte constitution of the cerebrospinal fluid (CSF) and influencing the cellular and/or transcellular transport of other ions, energy substrates and neurotransmitters (Blanco and Mercer, 1998; Skou, 1998; Blanco, 2005).

The enzyme is composed of two distinct polypeptides (Kyte, 1971), with equimolar stoichiometry (Craig and Kyte, 1980). The larger one is the α subunit, with an amino acid sequence (>1010 amino acids) that was first deduced from sheep kidney complementary DNA in 1985 by Jerry Lingrel's group (Shull et al., 1985). The α subunit has a mass of 110–112 kDa, with 10 transmembrane domains, mostly with protein resides in the cytoplasm side and with less than 10% exposed to the extracellular milieu (Blanco, 2005). The α subunit is also known as the catalytic or functional subunit, since it contains the binding sites for Na^+^ and K^+^, as well as for CTS. The α subunit is subject to transient phosphorylation and breaks down Mg^2+^-ATPase (Kyte, 1975; Blanco, 2005). The smaller β subunit is a type II (one transmembrane domain with intracellular N-terminal) glycoprotein of 302–304 amino acids that noncovalently binds to the α subunit (Shull et al., 1985). Although the α subunit can display independent ATP catalysis (Blanco et al., 1994), it is only when it is associated with the β subunit that normal pumping activity is achieved. The β subunit also influences the NKA kinetic characteristics, assisting the correct assembly and membrane delivery of the enzyme (chaperone function), as well as helping in cell adhesion and cell polarity (Blanco, 2005; Geering, 2008; Cereijido et al., 2012; Vagin et al., 2012). A third partner comprises the αβ protomer (formerly termed the γ subunit) (Forbush et al., 1978). This type I (one transmembrane domain with intracellular C-terminal) protein is small—around 65 amino acids, with a mass of 7 kDa—and belongs to the FXYD family of ion transport regulators. FXYD represents the signature motif found in all 7 members. FXYD2 is the original γ subunit, although all can interact with NKA (Geering, 2008). However, they are not necessary for enzymatic activity, modulating, usually decreasing, pump affinity for cations (Geering, 2006, 2008). The electrophoresis technique has unveiled puzzling pharmacological data that demonstrate differences in the potency and affinity of CTS for NKA, when NKA is derived from different tissues and species (Akera et al., 1972; Tobin and Brody, 1972; De Pover and Godfraind, 1976, 1979; Marks and Seeds, 1978; Noel and Godfraind, 1984). By the end of 1970's, Kathlyn Sweadner demonstrated in brain preparations of several mammalian species that two protein bands of slightly different molecular weights could be clearly defined, with the biochemical characteristics of NKA (Sweadner, 1979). The kidney α subunit co-migrated with the brain lower migrating (lighter) band in gel and was designated α (now α1), with the other brain band, heavier and not present in kidney, designated α(+) (Sweadner and Gilkeson, 1985). A third form was revealed from rat brain cDNA sequence analysis and named αIII (now α3), a major form in the central nervous system (CNS) (Shull et al., 1986; Schneider et al., 1988; Sweadner, 1989). α(+) was indeed a mixture of α2 and α3. Years later, a fourth α isoform was discovered and characterized (Shamraj and Lingrel, 1994; Woo et al., 1999; Blanco et al., 2000). All isoforms are encoded by different genes, have a high degree of sequence homology and are expressed in a cell/tissue-specific manner (Broude et al., 1989; Mobasheri et al., 2000; Blanco, 2005): α1 is expressed in all mammalian tissue, being virtually the only α isoform expressed in the kidney across species; α2 is found in striated and smooth muscles, adipose tissue, nervous tissue and some other tissues; α3 and α4 have a more restricted distribution, with the former being primarily found in nervous tissue, more specifically in neurons, where it may be considered a brain neuronal marker (Dobretsov and Stimers, 2005), and the latter is only expressed in the midpiece of the sperm, where it is important for sperm motility (Sanchez et al., 2006).

### A pump or a receptor: the janus face of an ATPase

NKA has been classically conceptualized as a pump, with its specific inhibition by CTS that increases intracellular Na^+^ and Ca^2+^ concentrations leading to cell ionic imbalance (Akera and Brody, 1977). This was challenged by a series of elegant studies headed by Zi-Jian Xie and Amir Askari 20 years ago. These researchers observed that OUA induced myocyte hypertrophy by activating proto-oncogenes and late response genes, with effects mediated by the Ras-Raf-ERK1/2-pathway (Peng et al., 1996; Huang L. et al., 1997; Kometiani et al., 1998). This was preceded by EGFR transactivation through the nonreceptor Tyr kinase, Src, steps not dependent on Ca^2+^(Haas et al., 2000). Such signal transducer pump effects arise from activity in restricted areas of the plasma membrane, within a subset of lipid rafts known as caveolae (Liu et al., 2003; Wang et al., 2004; Quintas et al., 2010). NKA (E1 state) can interact with Src at the kinase and SH3 domain with OUA, by arresting the E2 conformation of the ATPase, unleashing the kinase domain of Src, thereby inducing protein-protein signaling (Liang et al., 2006; Tian et al., 2006; Ye et al., 2013). As such, NKA-Src forms a binary receptor that transduces signals after CTS binding.

After the first reports on the novel signaling role of NKA, a number of different intracellular pathways involved in distinct cell fates has been unveiled, confirming NKA as more than an ion pump (Nesher et al., 2007; Cai et al., 2008; Riganti et al., 2011; Arnaud-Batista et al., 2012; Lucas et al., 2012) and helping to clarify how endogenous CTS can produce their effects *in vivo*, even at very low concentrations (Xie and Xie, 2005; Aperia et al., 2016).

### NKA mutations and diseases

Considering the importance of NKA in basic cellular functions, it has been suggested that NKA mutations affecting α1 (*ATP1A1*), α2 (*ATP1A2*), and α3 (*ATP1A3*) genes can contribute to the pathogenesis of several CNS diseases.

#### ATP1A1 mutations

Mutation in the *ATP1A1* gene leads to primary aldosteronism (Azizan et al., 2013), which is the main cause of secondary hypertension. Aldosterone production is elevated and non-suppressible by sodium loading (Duan and Mete, 2015). The mutation causes a decrease in NKA activity and in K^+^ affinity, consequently leading to an inappropriate cellular depolarization (Beuschlein et al., 2013). Primary aldosteronism can also be caused by mutations in ATP2B3 (Ca^2+^-ATPase), CACNA1D (Cav1.3), and KCNJ5 (K^+^ channel) (Azizan et al., 2013; Zennaro et al., 2015). In a study with 474 patients, *ATP1A1* mutation was found in 5.3% of the sample, although the relationship between the disorder and the *ATP1A1* mutation was only discovered recently, requiring further investigations as to the mechanism involved (Fernandes-Rosa et al., 2014).

#### ATP1A3 mutations

*ATP1A3* is only expressed in CNS neurons, mostly in the cerebellum and basal ganglia, key structures in the regulation of a range of functions, including motor activity, memory and spatial learning. The *ATP1A3* mutations are common in the conserved transmembrane or N-terminus domain of NKA and are related to rare disorders, such as rapid-onset dystonia-parkinsonism (RDP), alternating hemiplegia of childhood (AHC), and cerebellar ataxia, areflexia, pescavus, optic atrophy, and sensorineural hearing loss (CAPOS) syndrome. Although having many common features, these three diseases have quite distinct phenotypes (Sweney et al., 2015).

RDP (or DYT12) is a type of dystonia, being classed as a hyperkinetic movement disorder. RDP onset can be highly variable, occurring from 18 months to 55 years, suggesting considerable heterogeneity in its pathophysiology (Sweney et al., 2015). The main features are involuntary muscle contractions, abnormal posture and repetitive movements. RDP was first linked to *ATP1A3* mutations by De Carvalho Aguiar and colleagues in 2004 (de Carvalho Aguiar et al., 2004), having an autosomal dominant inheritance. However, this disorder can also be sporadic or not related to any mutation in *ATP13A* (Kabakci et al., 2005). Although some RDP symptoms resemble Parkinson's disease, with both disorders showing evidence of abnormal CSF dopamine metabolites, RDP patients are unresponsive to deep brain stimulation (Charlesworth et al., 2013) or to L-DOPA treatment (Asmus and Gasser, 2010). RDP differentiation from Parkinson's disease is based on: triggering by physical or emotional stress, abrupt onset, bulbar involvement and normal computed tomography in the striatum (Zanotti-Fregonara et al., 2008; Asmus and Gasser, 2010).

Twelve mutations are associated with RDP, each being related to different severity levels. RDP treatment is only symptomatic, mostly utilizing benzodiazepines (Sweney et al., 2015). The T613M mutation is the most common and with the most severe outcome. Psychiatric conditions, such as bipolar disorder and anxiety, seem to be related to RDP (Barbano et al., 2012). A growing variety of clinical presentations have been reported in association with these mutations, including episodes of flaccidity and lack of motion for hours leading to stiffness (Anselm et al., 2009) or delayed motor development and hypotonia that lead to a uncoordinated gait, as well as speech and swallowing difficulties in R756H and D823N mutations.

Recent studies on a family with RDP, where only women present with a symptomatic phenotype, indicate a new mutation that causes a deletion (c.443_445delGAG, p.Ser148del). A male member from the same family also carries the p.Ser148del mutation, but he does not have any symptoms, suggesting a reduced penetrance of this mutation (Wilcox et al., 2015). RDP patients are still commonly misdiagnosed, indicating the need to incorporate new features in order to make an appropriate diagnosis (Brashear et al., 2012).

The onset of motor symptoms in RDP patients occurs usually by 25 years of age, with initial symptoms occurring in the upper body. Following initial diagnosis, patients have been shown to have poor learning, memory and psychomotor speed vs. controls (Cook et al., 2014). This would seem to arise from α3 being highly expressed in all nuclei of the basal ganglia and cerebellum, which are important regions for the control of movement, and in the hippocampus, which is crucial to spatial learning and memory (Oblak et al., 2014). Experiments utilizing *in vitro* and *in vivo* models have investigated the alterations driven by these mutations, including the role of impaired NKA activity. A C-terminus protein mutation decreases Na^+^ affinity, although no abnormality in biogenesis or plasma membrane targeting was found. Also, a decreased survival in these neuronal cells was observed when they were treated with OUA (Blanco-Arias et al., 2009).

**Figure 1:**
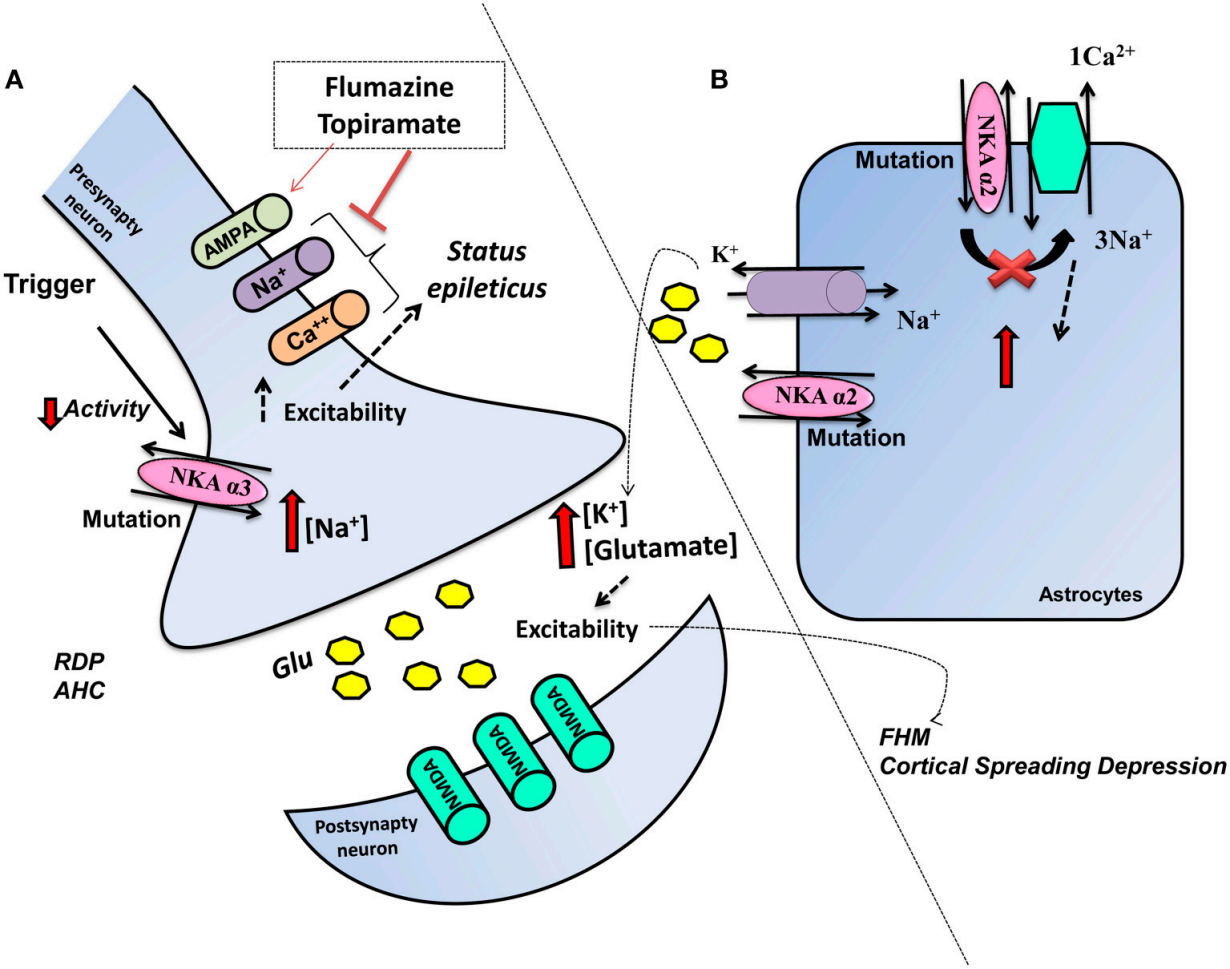
**Schematic representation of the influence of *ATP1A2* mutations in glia and *ATP1A3* mutations in neurons on glutamatergic system activity**. Mutations cause a dysfunctional NKA activity, **(A)** *ATP1A3* mutation in neurons decreases the NKA activity and increases intracellular Na^+^, which increases the cellular excitability, thereby affecting neuronal functions. Flumarazine and topiramate could be used as treatment in some cases. **(B)** *ATP1A2* in astrocytes results in change of metabolism, more K^+^, and glutamate in the extracellular space and rendering the CNS more vulnerable to migraine, seizures, and neurodegenerative process.

Other mutations were also tested in COS7 cells, with no phosphorylation from ATP and low Na^+^ affinity being detected in most mutations. Consequently, increased intracellular Na^+^ and decreased extracellular K^+^ were observed. Thus, the affinity of Na^+^ may be an important concern in RDP (Toustrup-Jensen et al., 2014) (Figure 1). In mutated α3 mice (Het mice), stress exposure was capable of causing motor and balance deficits characteristic of RDP (DeAndrade et al., 2011). These authors also showed significant alterations in monoamine metabolism and thermal sensitivity in this model (DeAndrade et al., 2011).

AHC is a neurodevelopmental disorder described initially in 1971 by Verret and Steele (1971). However, it was only in 2012 that AHC was discovered to be a heterozygous *ATP1A3 de novo* mutation (Heinzen et al., 2012; Rosewich et al., 2014). AHC patients present with an onset before the age of 18 months and following an environmental or physiological stimulus, which is also present in RDP. AHC patients present with motor delay and attacks of hemiplegia, as well as cognitive and intellectual deficits (Sweney et al., 2015). AHC disorder can be difficult to differential diagnosis especially in relation to familial hemiplegic migraine (FHM) and glutamate transport disease (Jen et al., 2005). Sleep can stop AHC attacks, although even after sleep, attacks can return. Avoidance of the possible triggers is also important to prophylaxis, whilst antiepileptic drugs should only be used in patients showing *status epilepticus* (Rosewich et al., 2014; Paciorkowski et al., 2015). Flunarizine is a Ca^2+^ and Na^+^ channel blocker, which was developed to treat migraine and is currently the main AHC treatment, although the mechanism of action is unknown. Similarly, topiramate's mode of efficacy is unknown and may include its inhibition of 2-amino-3-(3-hydroxyl-5-methyl-4-isoxazolyl) propionic acid (AMPA) receptors and carbonic anhydrase, thereby lowering extracellular pH (Hoei-Hansen Et al., 2014; Yang et al., 2014) (Figure 1).

Another strategy used in AHC patients is the ketogenic diet/modified Atkins diet, given that a family with the p.Asp923Asn mutation was initially misdiagnosed as GLUT1DS (glucose transporter deficiency syndrome), with 15 months of diet management leading to no further attacks. A ketogenic diet provides an alternative energy source for the brain and may decrease neuroexcitability (Roubergue et al., 2015). In some cases, when a patient has two mutations in SLC2A1 and *ATP1A3*, flunarizine provides an incomplete response, with concurrent use of a ketogenic diet improving the utility of flunarizine (Ulate-Campos et al., 2014). A patient with c.2401G>A; p.D801N mutation failed to respond to all the treatment strategies and only the use of a corticosteroid achieved complete remission (Wong and Kwong, 2015).

In Japan, the more severe phenotype arising from the E815K mutation was found to be more common than in other countries, occurring in 50% of investigated patients. The more severe phenotype evident in these mutations includes: earlier age of onset, more epileptic episodes and increased motor impairment (Ishii et al., 2013). Usually patients with no detectable mutation have less symptomatology (Hoei-Hansen et al., 2014), whilst an early onset of AHC symptoms seems positively correlated with a more severe clinical course (Viollet et al., 2015).

A Danish study showed bilateral episodes to be common, with hemiplegic episodes not always alternating. Consequently, the term AHC may not be the most appropriate (Hoei-Hansen Et al., 2014). A recent study of 155 patients showed 34 mutations to be related to AHC, with at least one being detected in 85% of patients. The three major mutations were found p.Glu815Lys, p.Asp801Asn, and p.Gly947Arg, with the first having the most severe phenotype, as indicated by greater intellectual impairment and motor disability. In contrast, p.Asp801Asn presents with a milder phenotype, whilst p.Gly947Arg appears to have the most favorable prognosis, in comparison to other mutations (Panagiotakaki et al., 2015).

Some mutations show a symptomatology that indicates a combination of RDP and AHC, including c.2600G>A, D923N and R756 (Rosewich et al., 2014). The E277K and D923N mutations have been described in both (Boelman et al., 2014). The difference between RDP and AHC may be due to different regions and cells affected by the mutations in ATP13A, with differential impacts arising from effects within a developmental context (Ozelius, 2012). Patients with epilepsy had an earlier onset, although no relationship with a specific mutation (Panagiotakaki et al., 2015). AHC patients have electrocardiogram and repolarization abnormalities compared to people with epilepsy, contributing to episodes of collapse unrelated to seizures, as well as to some premature deaths (Novy et al., 2014; Jaffer et al., 2015). Activated platelets from AHC patients have functional and structural abnormalities, with 93 proteins related to metabolism being differentially expressed in comparison to healthy controls. Cathepsin B was also more highly expressed, indicating an important role of apoptotic pathway activation in driving mutation-associated cell death (Di Michele et al., 2013). An AHC patient showing a mutation in SLC2A1 p.Gly18Arg, which leads to the loss of GLUT1 expression, was also diagnosed with hemiplegic migraine, which can be related to increases in cortex glutamate levels. As such, glutamate levels should also be investigated in mutation-associated AHC patients (Weller et al., 2015).

Mutations of *ATP1A3* that can be evident in AHC patients, have been tested in Sf9 cells. OUA binding, NKA activity and phosphorylation were measured for each mutant and were found to be abnormal in I274N, E815K, and G947R mutations. Some mutations showed normal bind to OUA, although no NKA activity or phosphorylation were found. Such differences may be associated with phenotype severity (Weigand et al., 2014). In a murine AHC model, the D801N mutant mouse (Mashlool, Mashl+/), a similar symptomatology is found as that evident in AHC patients, including abnormalities in gait and motor coordination, as well as spontaneous recurrent seizures and hemiplegia. The study of hippocampal slices showed increased excitability, which contributes to abnormal plasticity and a predisposition to more spontaneous seizures (Hunanyan et al., 2015).

Another animal model, the Myshkin (Myk) model, carries the I810N mutation, which presents with a reduced threshold for hippocampal seizures and neuronal loss. Although α3 is normally expressed, NKA activity is reduced with a consequent reduction in thalamocortical functional connectivity (Clapcote et al., 2009; Kirshenbaum et al., 2013). This murine model is also used as a model of bipolar disorder, with symptoms being improved by melatonin and physical exercise (Kirshenbaum et al., 2014). An α3 mutation is evident in the Atp1a3tm1Ling murine model, leading to a reduction in hippocampal α3 expression, thereby lowering NKA activity. Dystonia is only induced in this model after kainate injection into the cerebellum (Ikeda et al., 2013).

In 1996, CAPOS syndrome was described, with only the c.2452G> mutation in *ATP1A3* being found in four families studied. This is a gain-of-function mutation, which differs from the loss mutations evident in RDP and AHC (Demos et al., 2014). CAPOS is a rare syndrome associated with febrile illness, with each patient differing in regard to severity and outcome (Sweney et al., 2015). Recently, CAPOS syndrome has been shown to cause hemiplegic migraine in a single case study (Potic et al., 2015).

#### ATP1A2 mutations

Migraine is a brain disorder that affects over 10% of the population, characterized by a headache associated with neurological and autonomic symptoms. Moreover, it frequently occurs as a pulsating and unilateral headache, usually followed by phono/photophobia and nausea (Russell et al., 1994). Migraine is thought to be initiated by brain dysregulation, leading to activation and increased vulnerability of the trigeminovascular system, mainly the trigeminal nociceptive afferents innervating the meninges (Goadsby et al., 2002; Pietrobon and Striessnig, 2003; Pietrobon, 2005). Migraine disorders may be preceded, or not, by neurological symptoms that are often visual, and can therefore be classified as migraine with or without aura (Pietrobon and Striessnig, 2003; Pietrobon, 2005). Clinical studies indicate that migraine aura is related with a wave of cortical spreading depression (CSD). CSD is a strong wave of short duration of neuronal and glial cell depolarization, which extends over the cortex and is accompanied by long-lasting depression of neuronal activity (Lauritzen, 1994; Cutrer et al., 1998; Bowyer et al., 2001; Hadjikhani et al., 2001). Studies have shown a relationship between CSD and migraine aura in humans and animal models and its importance to trigeminal activation (Russell et al., 1994; Bowyer et al., 2001; Hadjikhani et al., 2001; Bolay et al., 2002; Goadsby, 2002; Dalkara et al., 2006; Zhang et al., 2010).

FHM is a special autosomal dominant subtype of migraine with aura, characterized by some degree of motor frailty or paralysis, often hemiparesis (Thomsen et al., 2002). Furthermore, severe cases have been associated with symptoms such as progressive cerebellar ataxia, coma, fever, and/or epileptic seizures (Ducros et al., 1997). FHM is a genetically heterogeneous disease, in which mutations can occur in the ion transportation genes *CACNA1A, ATP1A2*, and *SCN1A* (Rogawski, 2008), promoting the different types of familial migraines, FHM1, FHM2 and FHM3 (Russell et al., 1994; Pierelli et al., 2006; Deprez et al., 2008). FHM1 is induced by mutations in *CACNA1A* at chromosome 19p13, which encodes the Cav2.1 (P/Q-type) (Ophoff et al., 1996). FHM2 is occasioned by mutations in the *ATP1A2* at chromosome 1q23 (De Fusco et al., 2003). The last gene is *SCN1A*, encoding the α1 subunit of the neuronal sodium channel, the mutation in which leads to FHM3 (Dichgans et al., 2005). In this review, we describe only mutations in *ATP1A2*, as well as the role of glutamate in the development of FHM. FHM was the first human disease linked to mutations of the α2 catalytic subunit, which was first demonstrated in two Italian families (De Fusco et al., 2003). There is also a linkage between sporadic hemiplegic migraine (SHM) and mutations in α2 (Thomsen and Olesen, 2004; Kirchmann et al., 2006), with other brain disorders also associated with α2 mutations.

In the mouse, α2 is expressed in cardiac and skeletal muscle throughout life. However, in neurons, *Atp1a2* is only expressed during embryonic development (Moseley et al., 2003), being present in astrocytes and meningeal tissues in adulthood (McGrail et al., 1991; Watts et al., 1991; Peng et al., 1997). During neuronal activity, NKA is important for removing K^+^ from the synaptic cleft. Moreover, it is fundamental for the re-uptake of released glutamate from the extracellular space, since active transport of glutamate into neurons and astrocytes is regulated by Na^+^ and K^+^ gradients, emphasizing the importance of NKA in synaptic regulation (Ransom et al., 2000; D'Ambrosio et al., 2002; Jorgensen et al., 2003). *Atp1a2*-deficient embryos have impaired neurotransmitter clearance, as well as potentiated neural excitation and cell death, specifically in the amygdala and piriform cortex. Consequently, heterozygous adult mice show increased levels of anxiety/fear behaviors, concurrent to raised levels of c-Fos and increased neuronal activity in the piriform cortex and amygdala after fear stimuli. This indicates a role for α2 in regulating not only neural activity (Ikeda et al., 2003), but also affective processing, including in the role of the amygdala in cortex development.

Kawakami et al. (2005) reported that heterozygous *Atp1a2* mice, $\alpha_{2}^{+∕-}$KOE_21_, show spontaneous epileptic seizures. In addition, their data demonstrated neuronal apoptosis in the piriform cortex and amygdala (Ikeda et al., 2003), being a possible explanation as to the manifestation of epilepsy in 20% of FHM2/SHM families (Bianchin et al., 2010). These epileptic seizures arise when neurons situated in temporal lobe regions, including the amygdala, piriform cortex and hippocampus become hyperexcitable (Aroniadou-Anderjaska et al., 2008). However, histological changes were not observed in the piriform cortex and amygdala in the brains of $\alpha_{2}^{-∕-}$KOE_21_ knockout mice (Ikeda et al., 2004). A colocalization of the NKA α2 with NCX occurs in astrocyte cultures, suggesting a central NKA role in the modulation of intracellular Ca^2+^ of the endoplasmic reticulum (Juhaszova and Blaustein, 1997). In addition, high levels of intracellular and intra-endoplasmic reticulum Ca^2+^ ions were detected in cultured astrocytes from *Atp1a2*-deficient mice (Golovina et al., 2003), linking NKA α2 to the manifold consequences of Ca^2+^ dysregulation.

Mutation in astrocyte NKA α2 facilitates both neuronal paroxysmal depolarizing shifts and hyper-excitability, triggering seizures as well as CSD induction and propagation in FHM/SHM patients (Pietrobon, 2005). Bolay et al. (2002), using an animal model of migraine, observed that CSD activates a N-methyl-D-aspartate (NMDA)-dependent pathway leading to an activation of nociceptive trigeminal afferents in the meninges, resulting in headache. Additionally, a specific role for NKA in glutamate clearance during synaptic transmission has also been established, with NKA being stimulated by glutamate in astrocyte cultures (Pellerin and Magistretti, 1997). Furthermore, a colocalization and functional coupling between NKA and glutamate transporters has also been demonstrated (Rose et al., 2009), with α2 having a similar perisynaptic glial localization to the glutamate transporters GLAST and GLT-1in the rat somatosensory cortex (Cholet et al., 2002; Rose et al., 2009). As such, inefficient astrocyte glutamate reuptake resulting in increased cortical excitatory neurotransmission, specifically as driven by NMDA receptor-dependent transmission during high-frequency action potential sequences, may enhance the sensibility to CSD in FHM2 (Tzingounis and Wadiche, 2007). Wider ionic dysregulation may also be evident, with the increase of extracellular K^+^ levels resulting from the impaired clearance by NKA, and an increase in the intracellular Na^+^ resulting in an elevation in the intracellular Ca^2+^ level through the NCX (Pietrobon, 2005), contributing to wider central disruption. Moreover, recent data indicates that impaired glutamate clearance by astrocytes, and involving the α2, is an important molecular mechanism of FHM2 (Figure 1). This *in vitro* study, in a hippocampal mixed astrocyte and neuronal culture derived from homozygous $\alpha_{2}^{\text{G}301\text{R}∕\text{G}301\text{R}}$ E17 embryonic mice, demonstrated reduced glutamate uptake due to the loss of functional NKAα2 in astrocytes. Interestingly, *in vivo* data in the same study showed a relationship between the glutamate system and the female sex hormone in psychiatric manifestations of FHM2. Furthermore, higher glutamate levels were evident in different brain regions from adult female $\alpha_{2}^{+∕G301R}$ mice, compared to male mice (Bøttger et al., 2016).

In support of a role for glutamatergic dysregulation in migraine, MTDH, a regulator of glutamate transporters, associates with the frequent form of migraine with aura. Furthermore, a mutation in the SLCA3 gene that encodes the glial excitatory amino acid transporter type 1 (EAAT1) has also been found, leading to neuronal hyper-excitability and consequent hemiplegia, as well as seizures and episodic ataxia, which arise as a consequence of impaired glutamate uptake (Jen et al., 2005).

Recent studies have demonstrated a link between long-term memory formation in the rat hippocampus and the astrocyte-neuron lactate transport (Suzuki et al., 2011). Furthermore, there is a relationship between NKA and the astrocyte specific glutamate-dependent transport of lactate (Kleene et al., 2007). This interesting finding may contribute to the pathophysiological processes underlying language disorder, cognitive impairment and mental retardation evident in FHM2/SHM patients (Le Pira et al., 2004; Kalaydjian et al., 2007). As such, evidence suggests that the central glutamatergic system has an important role in driving the consequences of *ATP1A2* mutations and should also be more explored in *ATP13A* mutations, due to the diagnostic confusion and overlap with glutamate transporter diseases.

### Glutamatergic signaling: a brief introduction

Glutamate is an amino acid, which is considered the main central excitatory neurotransmitter, being highly abundant in the majority of animals, especially in the cortex, hippocampus and caudate nucleus (reviewed in Erecinska and Silver, 1990). Glutamate's cellular actions are mediated through the activation of metabotropic and ionotropic receptors. The ionotropic receptors are divided according to their affinities for specific agonists, namely NMDA, AMPA, and kainate (McLennan, 1981). The glutamatergic metabotropic receptors were first investigated in late 1980's (Nicoletti et al., 1986). There are eight types of metabotropic receptors divided into three groups (Groups I, II, and III) depending on DNA sequence and the signaling pathway they activate (reviewed in Wieronska et al., 2016).

Glutamate has many roles, including being part of intermediary metabolism in the brain (Krebs, 1935; Berl et al., 1961) as well as acting as a precursor for different compounds, such as the inhibitory neurotransmitter gamma-aminobutyric acid (GABA) (Roberts and Frankel, 1950). By acting through metabotropic and/or ionotropic receptors, glutamate is also an important element in a variety of core processes such as learning and memory, partly by regulating synaptic plasticity *via* long-term potentiation (LTP) (Bliss and Collingridge, 1993; Vickery et al., 1997), long-term depression (LTD) (Dudek and Bear, 1992; Yokoi et al., 1996; Nicoll et al., 1998; Dong et al., 2013) or both (Manahan-Vaughan, 1997; Huang L. Q. et al., 1997; Wang et al., 2015). The hippocampus and cortex are the major brain regions investigated in studies of cognition, with these regions also showing the highest concentrations of brain glutamate. Glutamate is crucial to many other brain processes, including motor behavior (Conquet et al., 1994; Pekhletski et al., 1996; Coesmans et al., 2003; Talpalar and Kiehn, 2010; Ohtani et al., 2014; Guimaraes et al., 2015), as well as in brain development (Deisseroth et al., 2004; Poulsen et al., 2005; DeSilva et al., 2012; Yamasaki et al., 2014; Jantzie et al., 2015).

Abnormalities in glutamate function can arise from alterations in a variety of factors, including: glutamate transporters and glutamatergic receptors, as well as glutamate metabolism and synthesis. Also, changes in the levels of glutamate's precursor, glutamine, may drive alterations in the extracellular glutamate concentration. Inflammation is linked to a host of medical conditions, with effects linked to sustained glutamate elevations (Takaki et al., 2012) or changes in glutamatergic receptor expression (Drouin-Ouellet et al., 2011). Increased levels of glutamate contribute to inflammatory process, as indicated by the efficacy of glutamatergic receptor antagonist in reducing inflammation (Bonnet et al., 2015).

Lack of glutamate could lead to memory deficits, given that LTP in some brain areas is NMDA receptor-dependent (Malenka and Bear, 2004). However, excess glutamate can be excitotoxic to neurons. A number of studies show a strong correlation between abnormal glutamatergic signaling pathway and neurodegenerative/psychiatric diseases, including Alzheimer's disease (Takahashi et al., 2015; Haas et al., 2016), Parkinson's disease (Alvarsson et al., 2015; Morin et al., 2015), Huntington's disease (Behrens et al., 2002; Estrada-Sánchez et al., 2009), amyotrophic lateral sclerosis (Plaitakis et al., 1988; Veyrat-Durebex et al., 2015), epilepsy (Wong et al., 2015), ischemia (Dhami et al., 2013), migraine (Peres et al., 2004; Campos et al., 2013), schizophrenia (Spangaro et al., 2012; Matsuno et al., 2015), depression (Berman et al., 2000; Duric et al., 2013; Peng et al., 2016), addiction (Aitta-aho et al., 2012; Perry et al., 2016), and autism spectrum disorder (Bristot Silvestrin et al., 2013).

### NKA and glutamatergic signaling

In the mammalian CNS, astrocytes are the most abundant glial cells. Astrocytes show two different NKA α isoforms, α1 and α2, with α1 being ubiquitously expressed (Geering, 2008). Under physiological conditions, during excitatory synaptic activity, astrocytes are responsible for glutamate uptake from the synaptic space, keeping extracellular levels of glutamate at nanomolar concentrations, and thereby avoiding the harmful consequences of glutamate receptor over-stimulation (Rothstein et al., 1996; Herman and Jahr, 2007; Kirischuk et al., 2012; Illarionova et al., 2014; Zhang et al., 2016). Astrocytes are therefore considered significant regulators of glutamatergic synaptic transmission (Amara and Fontana, 2002; Zhang et al., 2016). The glial glutamate transporters (GluTs) that mediate glutamate uptake rely on the NKA electrochemical gradient (Levy et al., 1998; Anderson and Swanson, 2000; Chatton et al., 2000; Kanner, 2006). The family of excitatory amino acid transporters (EAAT) are composed of the glutamate transporters, with GLAST (glutamate/aspartate transporter or EAAT1) and GLT-1 (glutamate transporter 1, or EAAT2) being considered the most important (Tanaka, 2000; Zhang et al., 2016) and the predominant type of GluT in adult astrocytes (Rothstein et al., 1996; Dunlop, 2006; Matos et al., 2013). As such, an alteration in the NKA ionic and electrochemical gradient is intimately associated with astrocyte regulation of glutamatergic activity.

In the cell, ATP depletion can lead to a reversal (Longuemare et al., 1999), or inhibition, of glutamate uptake (Sheean et al., 2013; Shan et al., 2014), as shown by inhibitory doses of OUA, which impairs glutamate transport (Pellerin and Magistretti, 1997; Rose et al., 2009; Genda et al., 2011) and leads to the redistribution and clustering of GluTs (Nakagawa et al., 2008; Nguyen et al., 2010). Some studies indicate a co-localization and physical interaction between the α2 subunit and GluTs (Cholet et al., 2002; Rose et al., 2009; Genda et al., 2011; Matos et al., 2013; Illarionova et al., 2014), suggesting NKA regulation of GluTs by direct contact, as well as *via* the electrochemical gradient.

Elevated glutamate concentrations (up to 50 μM) may stimulate NKA activity (Gegelashvili et al., 2007). This was observed in a study of human fetal astrocytes, which showed that during synaptic activity, when glutamate is elevated, there is an increase in cell membrane expression of the NKA FXYD2/γ subunit and GLAST, suggesting a possible interaction between them (Gegelashvili et al., 2007; Nguyen et al., 2010; Zhang et al., 2016). Furthermore, the inhibition of Src in forebrain synaptosomes with PP2 or SU6656, as well as the inhibition of NKA by OUA, both decreased glutamate transport activity to the same level (Rose et al., 2009), suggesting the existence of a possible protein complex formed between NKA/Src/GluT (Rose et al., 2009). Moreover, adenosine can modulate glutamate uptake through activation of astrocyte adenosine A_2A_ receptors (A_2A_Rs), which can interact with the α2 isoform in the adult mouse brain, leading to the inhibition of astrocyte glutamate uptake (Matos et al., 2013). This data strengthens the idea of a macromolecular complex in astrocytic membranes encompassing A_2_ARs, α2 and GLT-I (Matos et al., 2013; Reinhard et al., 2013), with parallels in neurons. In 2009, Rose and colleagues showed that in the rat hippocampus GLT-1b isoform is co-expressed with α2 in astrocytes, whereas GLT-1a isoform was co-expressed with α3, a neuronal specific isoform (Chen et al., 2004; Bassan et al., 2008; Furness et al., 2008; González-González et al., 2008; Rose et al., 2009; Petr et al., 2015).

Notwithstanding the existence of a well described physical interaction between α2 and GluTs (Cholet et al., 2002; Porras et al., 2008; Rose et al., 2009; Genda et al., 2011; Bauer et al., 2012; Matos et al., 2013; Rose and Chatton, 2016), the substantial Na^+^ influx that is driven into astrocytes during synaptic activity has been related to a possible functional interaction, whereby astrocytes increase the local availability of metabolic substrates (Rose and Chatton, 2016). During glutamate stimulation, there is a dramatic reduction in neuronal ATP mediated by NMDA receptor activation followed by an increase in NKA activity (Foo et al., 2012).

In the 1990's, Inoue and Matsui observed an increase in K^+^ uptake by α2 and α3 after stimulation of rat embryonic neurons with glutamate (100 μM) (Inoue and Matsui, 1990). Similar results were observed some years later in Wistar adult rat slices (Nathanson et al., 1995; Munhoz et al., 2005) by Munhoz et al. (2005), which described a pathway whereby glutamate stimulation of NMDA receptor-NOS leads to increased NKA activity, possibly due to increased cyclic GMP (cGMP) synthesis. Glutamate-induced Ca^2+^ influx leads to the activation of neuronal nitric oxide synthase (nNOS) and increased production of nitric oxide (NO). NO, by activating soluble guanylylcyclase (sGC) enzyme, increases concentrations of the second messenger cGMP, thereby up-regulating cGMP-dependent protein kinase (PKG). PKG modulates NKA, altering the activity of NKA isoforms, both centrally and peripherally (McKee et al., 1994; de Oliveira Elias et al., 1999; Munhoz et al., 2005; Scavone et al., 2005). Accordingly, a strong correspondence occurs between pharmacologically induced cGMP and NKA modulation in the cerebellum, reinforcing the role of the glutamate-NO-cGMP pathway in NKA regulation (Nathanson et al., 1995).

In 1999, Inoue and colleagues showed that when primary neuronal cultures from rat embryos were treated with three typical agonists of ionotropic glutamate receptors, namely kainic acid (KA), AMPA and NMDA, a remarkable increase in the activity of α2 and α3 isoforms and a slight decreased activity of α1 was observed, suggesting that glutamate effects on NKA may be mainly mediated by ionotropic glutamate receptors (Inoue et al., 1999; Kawamoto et al., 2012), with the neuronal NKA isoforms differing in physiological functions and playing a crucial role in restoring ion gradients after neuronal excitation (Inoue et al., 1999). Meanwhile, Brines and Robbins (1992) observed that prolonged inhibition of α2 and α3 could lead to the potentiation of glutamate neurotoxicity, with NKA activity also regulating the cell surface expression and turnover of AMPA receptors (Zhang et al., 2009). Interestingly, when primary cultured cerebellar neurons were treated with 0.1 μM glutamate a 37% increase in NKA activity was observed, while a concentration of 100 μM glutamate was able to increase NKA activity by 85%. However, when pre-treated with the NMDA receptor antagonist, MK-801, NKA activity increase was abrogated, suggesting that glutamate-driven NKA activation is mediated by NMDA receptors (Marcaida et al., 1996). Once NMDA receptors are activated, the subsequent Ca^2+^ influx can activate calcineurin, a calcium/calmodulin-dependent protein phosphatase (Marcaida et al., 1996; Rambo et al., 2012; Unoki et al., 2012; de Lores Arnaiz and Bersier, 2014), which, through NKA dephosphorylation, restores NKA enzyme activity (Bertorello et al., 1991; Aperia et al., 1992; Marcaida et al., 1996). It is well proven that protein kinase C (PKC) phosphorylates the α subunit, leading to a decrease in its enzyme activity (Bertorello and Aperia, 1989; Fukuda et al., 1991; Beguin et al., 1994; Marcaida et al., 1996; Cheng et al., 1997; Chibalin et al., 1998; Nishi et al., 1999; Taub et al., 2010; Liu et al., 2016). Calcineurin can desensitize NMDA receptors (Lieberman and Mody, 1994) *via* the C-terminus of the NR2A subunit (Krupp et al., 2002) and regulates calmodulin effects, reducing the open time of the channel (Rycroft and Gibb, 2004).

During learning processes PKC is active and phosphorylates some Src family kinases (SFK), which in turn induces tyrosine phosphorylation of NMDA receptors, leading to a rapid up-regulation of NMDA receptor membrane expression (Groveman et al., 2012). Some studies have proposed that Src protein forms a complex with the NMDA receptor through the binding of its SH2 domain with the N-terminal region of postsynaptic density protein 95 (PSD-95). Furthermore, PSD-95 seems to negatively regulate Src (Kalia et al., 2006; Groveman et al., 2012). Indeed, co-activation of mGluR5 and the NMDA receptor with low concentrations of their agonists (CHPG and NMDA, respectively), in rat hippocampal slices, leads to the phosphorylation of NR2B (Tyr1472) by Src kinase (Sarantis et al., 2015), in turn increasing NMDA-mediated excitability.

It is important to note that NKA acts as a specific receptor for CTS. The interplay between the NKA and NMDA receptors is proposed to arise from the initial administration of CTS leading to conformational changes that enhance NMDA subunit expression as a compensatory mechanism (de Lores Arnaiz and Bersier, 2014). OUA is also able to regulate NMDA binding and receptor activity in the adult rat brain (Reines et al., 2001, 2004; Bersier and Rodríguez de Lores Arnaiz, 2009), in a concentration-dependent manner (Bersier and Rodríguez de Lores Arnaiz, 2009). Akkuratov et al. (2015), in rat cerebellar neurons, showed that both α1 and α3 subunits can functionally interact with NMDA receptors whereas Sibarov and colleagues demonstrated by patch-clamp and Ca^2+^ imaging experiments that rat cortical neurons treated with 30 μM NMDA or KA with 1 nM OUA or digoxin for 4 or 6 h, were protected from apoptosis (Sibarov et al., 2012; Abushik et al., 2013). Similar protection was observed *in vivo* (Golden and Martin, 2006). One possible explanation is that an up-regulation of the plasma membrane NCX leads to more Ca^2+^ extrusion preventing the increase of sEPSC frequency, which is typically found in excitotoxicity process. This up-regulation of NCX activity seems to be due to an interaction between NKA and NCX (Sibarov et al., 2012).

Furthermore, a study published in 2013, in primary cerebellar cell culture treated with OUA in different concentrations (1, 10, and 100 mM) for different times (1, 2, and 4 h), suggested nuclear factor-kappaB (NF-κB) activation in a time and concentration-dependent manner. However, this effect was not observed when neurons were pre-treated with MK-801, PP1 (Src-family tyrosine kinase inhibitor), manumycin A (farnesyltransferase inhibitor), and PD98059 (mitogen-activated protein kinase (MAPK) inhibitor). As such, this study described a pathway whereby OUA activates NF-κB through the NMDA receptor-Src-Ras-MAPK pathways (de Sá Lima et al., 2013). Strong evidence indicates that NMDA can activate NF-κB in the rat hippocampus (Lipsky et al., 2001; Kawamoto et al., 2012), with intra-hippocampal injection of 1 μM NMDA 1 h before the injection of 10 nM OUA potentiating NF-κB activity (Kawamoto et al., 2012). This activation was reduced when cells were pre-treated with an NMDA receptor antagonist (MK-801), suggesting that NF-κB activation by OUA, in part, arises from NMDA receptor modulation (Kawamoto et al., 2012). Overall, the above results indicate that OUA, through the action of NKA as a pump or as a receptor, can modulate many signals important in neurotransmission.

### NKA-glutamatergic signaling: aging and neurodegenerative diseases

**Figure 2:**
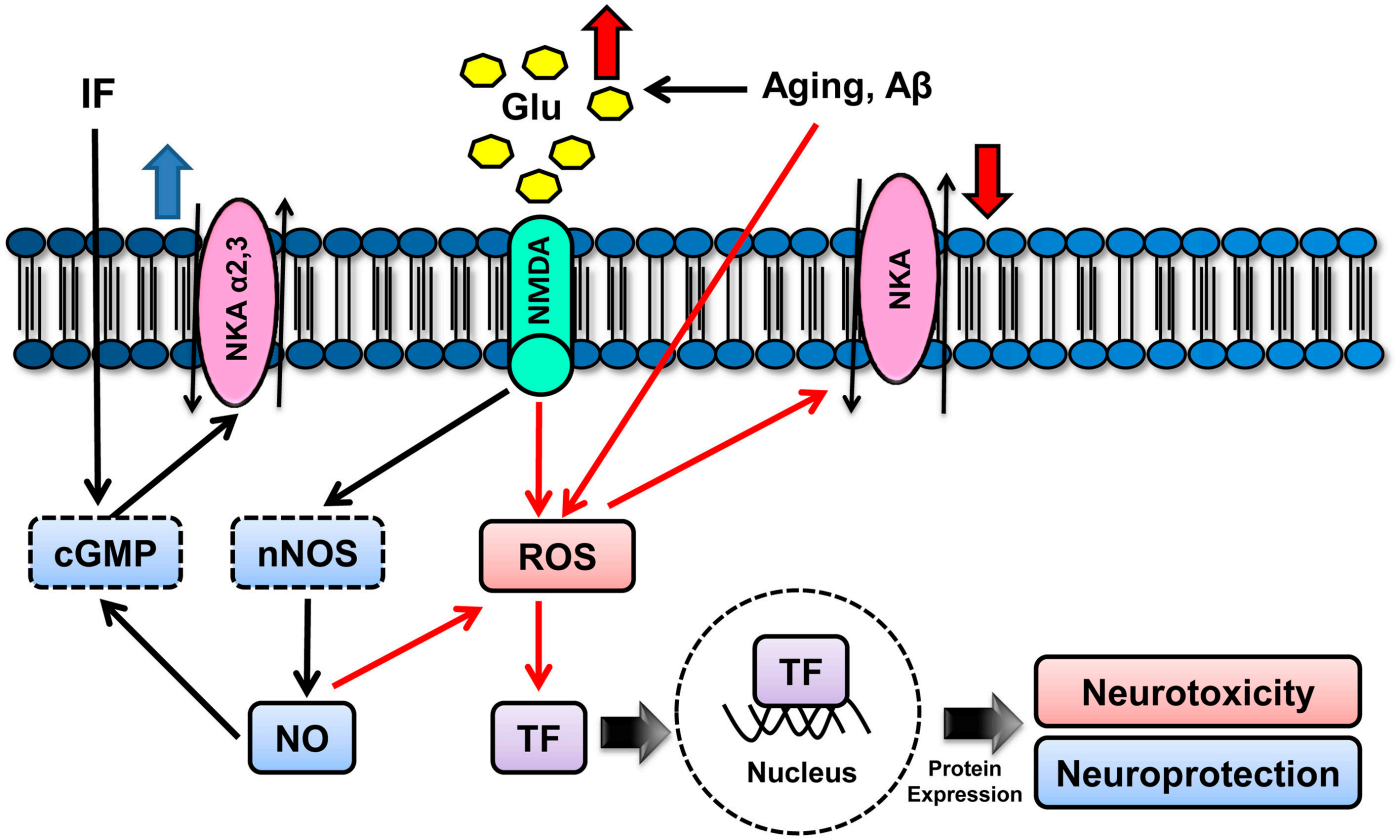
**Schematic model for molecular mechanisms underlying aging modulatory effects on NKA isoforms**. Aging can either increase the production of ROS, such as superoxide radical and hydrogen peroxide, or induce NO^•^ release by impairing Ca^2+^ homeostasis and subsequently increasing intracellular Ca^2+^ (nNOS-mediated NO^•^ production). NO^•^ is a free radical and can generate peroxynitrite, which may cause neurotoxicity by lipid peroxidation, mitochondria disruption, mutations of DNA and proteins, apoptosis and impairment of α1 activity. Strategies that induce NMDA activation can also activate the cGMP pathway, which, in turn, may lead to neuroprotective signaling, partly by upregulating α2 and α3 (cGMP, cyclic GMP; GLU, glutamate; NKA, Na^+^,K^+^-ATPase).

Several studies have shown age-related modifications in the NMDA receptor-NO-cGMP cascade in the CNS (Peterson and Cotman, 1989; Tamaru et al., 1991; Wenk et al., 1991; Vallebuona and Raiteri, 1995; Wardas et al., 1997; Ossowska et al., 2001; Chepkova et al., 2015). In addition, the aging process is associated with a progressive decline in NKA activity, both centrally and peripherally (Scavone et al., 2000; Kawamoto et al., 2008; Vasconcelos et al., 2015). In the CNS, this reduction is due to the modulation of α2 and α3 isoforms with no change in α1 and Mg^2+^-ATPase activities, as reported in the rat cerebellum and hippocampus (Kawamoto et al., 2008; Vasconcelos et al., 2015). As no differences in the α2 and α3 protein levels are evident, the age-related decrease in NKA activity is linked to modifications in glutamate-cGMP-PKG pathway (Kawamoto et al., 2008). The age-related failure of NKA modulation by glutamate through cGMP-PKG signaling may be connected to ionic disturbances during senescence, which may predispose to neurodegenerative diseases (Scavone et al., 2005). Interestingly, while inflammation, closely associated with aging and neurodegeneration, worsens aging-associated glutamate signaling and NKA defects, intermittent fasting counteracts this effect (Vasconcelos et al., 2015) (Figure 2).

DNA repair defects (Garinis et al., 2008; Schumacher et al., 2008; Végh et al., 2012), and consequent mitochondrial dysfunction (Zahn et al., 2007; Yankner et al., 2008), are considered important causes of aging and, in turn, with neurodegenerative disorders (Lin and Beal, 2006; Johri and Beal, 2012). Likewise, ROS-induced mitochondria impairment may contribute to the etiology of neurodegenerative disorders (Lin and Beal, 2006). Compromised mitochondria lead to impaired energy supply, producing less ATP and even more free radicals, as a consequence of inefficient oxidative phosphorylation, which, in turn, can directly or indirectly affect NKA activity (Nathanson et al., 1995). In support of this, a number of studies show the α subunit to be sensitive to free radical attack (Kim and Akera, 1987; Mense et al., 1997; Xie and Xie, 2005), with the oxidized α subunit being degraded (Zolotarjova et al., 1994; Thevenod and Friedmann, 1999). Furthermore, age-related increases in the levels of the free radical superoxide (${\text{O}}_{2}^{-}$) lead to ONOO^−^ formation *via* reaction with the NO generated by glutamate signaling (Wink et al., 1996). ONOO^−^ also impairs NKA activity (Boldyrev et al., 1997; Sato et al., 1997; Muriel and Sandoval, 2000; Kocak-Toker et al., 2005; Reifenberger et al., 2008). NKA inhibition also increases neuronal susceptibility to glutamate excitotoxicity, at least in part by reducing the Na^+^ gradient necessary for glutamate transport and clearance from the synapse cleft, leading to a positive feedback loop (Lees et al., 1990; Brines and Robbins, 1992). Both cGMP and PKG are well-established as important mediators of LTP, which NKA also modulates (Stevens and Wang, 1993; Zhuo et al., 1994; Nathanson et al., 1995). Moreover, NKA impairment downregulates the synaptic AMPA receptor, contributing to synaptic transmission defects, and consequently cognitive decline (Zhang et al., 2009). Since cognitive impairment is a hallmark of aging and neurodegenerative diseases, NKA-mediated glutamate signaling dysfunction may be an important contributory factor to neurodegenerative disorders (Végh et al., 2012).

EAAT downregulation has been associated with many neurodegenerative diseases (Sheldon and Robinson, 2007). As described above, the resulting elevation in glutamate levels in the synapse cleft and consequent excitotoxicity may interfere in NKA functioning by increasing NO release and free-radical production (Dawson et al., 1991).

Glutamatergic signaling dysfunction and its associated consequences are critical contributors to the pathophysiological underpinnings of Alzheimer's disease (AD) (Lewerenz and Maher, 2015). AD is the most frequent form of dementia, being characterized by memory and cognitive impairments. The AD brain shows two classical changes: increased Aβ peptide deposition in senile plaques; and tau hyperphosphorylation that leads to neurofibrillary tangles (Khachaturian, 1985; Mattson, 2004). AD is also associated with increased ROS production (Butterfield et al., 2001), compromised glutamate clearance (Lauderback et al., 2001; Vitvitsky et al., 2012) and increased immune-inflammatory activity, in association with raised levels of glutamate, which regulates tryptophan catabolites (Maes and Anderson, 2016).

ATPase activities play a key role in protecting neurons from excitotoxic insults (Hattori et al., 1998). Hattori and colleagues showed reduced NKA activity in postmortem AD patients' brains (77.2% of the controls) with no change in Mg^2+^-ATPase activities (Hattori et al., 1998). The protein levels of alpha and beta subunits were also decreased in AD brains (Hattori et al., 1998). However, although this study showed a clear reduction of NKA activity in the frontal lobes of AD brains (Hattori et al., 1998), a previous study found no change in NKA enzyme activity in 4 AD brains vs. 5 control brains (Liguri et al., 1990).

In an *in vitro* study, the acute administration of Aβ_25−35_ or Aβ_1−40_ oligomers in rat hippocampal neurons resulted in lower NKA activity (Mark et al., 1995), suggesting a role of Aβ toxicity in the NKA activity impairment. Also, a disruption in NKA activity leads to Na^+^ intracellular accumulation, increasing the influx of calcium through voltage-dependent calcium channels (Mark et al., 1995), suggesting an NKA role in AD cellular toxicity and apoptosis (Hattori et al., 1998). In an AD model, the APP+PS1 transgenic mouse, a decrease in hippocampal NKA activity and protein levels is also evident (Dickey et al., 2005), further implicating a role for Aβ regulation of NKA in AD.

Supporting the NKA dysfunction in AD, Vitvitsky and colleagues showed an imbalance of Na^+^ and K^+^ ion concentrations in both postmortem AD brain tissue and *in vitro* primary astrocytes treated with Aβ_25−35_ or Aβ_1−40_peptides (Vitvitsky et al., 2012). The electrochemical Na^+^ gradient maintained by NKA is the driving force for glutamate re-uptake by EAATs (Rose et al., 2009). In the astrocyte cell culture, ion homeostasis disruption was associated with reduced levels of NKA and Na^+^-dependent EAATs (Vitvitsky et al., 2012). Furthermore, homocysteine, a protein involved in many CNS disorders, including Parkinson's disease (Kuhn et al., 1998) and AD (Gallucci et al., 2004), was shown to reduce NKA activity and consequently impair glutamate reuptake in the rat hippocampus (Machado et al., 2011). Hence, NKA impairment associated with glutamate signaling imbalance may contribute to the pathophysiology of AD and other neurodegenerative diseases by significantly deregulating membrane transport, brain electrophysiological activity and other important cellular processes (Vitvitsky et al., 2012). Huntington's disease (HD) is another neurodegenerative disorder associated with cognitive decline and synapse impairment (DiFiglia et al., 1995; Smith et al., 2005; Rozas et al., 2010; Nithianantharajah and Hannan, 2013; Valencia et al., 2013). Many studies show glutamate signaling to be defective in HD, which is proposed to play a role in its pathophysiology (Coyle and Schwarcz, 1976; Beal et al., 1986; Zeron et al., 2002; Shehadeh et al., 2006). In Hdh^140Q∕140Q^, a knock-in mouse model of HD, a reduction of NKA and glutamate transporters, VGlut1 and Vglut2, by more than 30% is evident at 12 months vs. controls (Valencia et al., 2010, 2013).

Amyotrophic lateral sclerosis (ALS) is an age-related fatal disease characterized by progressive motoneuron degeneration. The loss of Cu/Zn superoxide dismutase 1 (SOD1) activity, an antioxidant enzyme, following increased oxidative stress, is widely thought to have a role in the etiology and/or course of ALS (Julien, 2001). Other possible contributing factors include increases in iNOS (Almer et al., 1999), glutamate excitotoxicity due to EAATs dysfunction (Rothstein et al., 1995; Bruijn et al., 1997; Trotti et al., 1999) and mitochondrial defect (Beal, 1995). Importantly, Ellis et al. showed that the NO-sGC-cGMP pathway was significantly impaired in the transgenic SOD1 ALS murine model, with this cascade being unable to modulate NKA activity (Ellis et al., 2003). Furthermore, in this study, a marked reduction in NKA activity accompanied by a decrease in the protein levels of the α subunits was evident. As to whether such changes are causal or a consequence of other pathophysiological processes in ALS requires further investigation (Ellis et al., 2003).

## Conclusions and future perspective

NKA is present in the membranes of most eukaryotic cells and acts as ion pump. NKA can be modulated by hormones and neurotransmitters, allowing these factors to regulate NKA's diverse important effects, including in the regulation of neurotransmission. NKA can also act as a receptor for steroids, as typified by the effects of OUA, with this being another mechanism whereby it contributes to the regulation of many essential cellular functions, centrally and peripherally. Changes in NKA activity, as occurs in aging as well as when arising from mutations, will play a role in a host of CNS diseases, partly via deficits in energy and glutamatergic regulation. Central energy deficiency has been proposed to be a key factor in many, currently poorly managed, neurodegenerative diseases, which are linked to changes in brain metabolism and increased levels of apoptosis. Given that NKA is fundamental for optimizing central synaptic functioning, as well as energy regulation, its further study, including its environmental, epigenetic and genetic regulation, are likely to be important in the development of new pharmacological and non-pharmaceutical treatments for a host of medical conditions that are currently poorly managed, as well as for the study and regulation of aging *per se*.

## Author contributions

Conceived and designed the manuscript: EK, LQ, and CS. Wrote the manuscript: AO, AV, PK, JL, and LQ have contributed equally to this work. Final revision: LQ, EK, CS, AO, PK, AV, JL.

### Conflict of interest statement

The authors declare that the research was conducted in the absence of any commercial or financial relationships that could be construed as a potential conflict of interest.
